# Prioritizing the mHealth Design Space: A Mixed-Methods Analysis of Smokers’ Perspectives

**DOI:** 10.2196/mhealth.5742

**Published:** 2016-08-05

**Authors:** Andrea Lisabeth Hartzler, June BlueSpruce, Sheryl L Catz, Jennifer B McClure

**Affiliations:** ^1^ Group Health Research Institute Seattle, WA United States; ^2^ University of California Davis Betty Irene Moore School of Nursing Sacramento, CA United States

**Keywords:** mobile health, human-centered design, human-computer interaction, smartphone, smoking cessation, telemedicine, software design

## Abstract

**Background:**

Smoking remains the leading cause of preventable disease and death in the United States. Therefore, researchers are constantly exploring new ways to promote smoking cessation. Mobile health (mHealth) technologies could be effective cessation tools. Despite the availability of commercial quit-smoking apps, little research to date has examined smokers’ preferred treatment intervention components (ie, design features). Honoring these preferences is important for designing programs that are appealing to smokers and may be more likely to be adopted and used.

**Objective:**

The aim of this study was to understand smokers’ preferred design features of mHealth quit-smoking tools.

**Methods:**

We used a mixed-methods approach consisting of focus groups and written surveys to understand the design preferences of adult smokers who were interested in quitting smoking (N=40). Focus groups were stratified by age to allow differing perspectives to emerge between older (>40 years) and younger (<40 years) participants. Focus group discussion included a “blue-sky” brainstorming exercise followed by participant reactions to contrasting design options for communicating with smokers, providing social support, and incentivizing program use. Participants rated the importance of preselected design features on an exit survey. Qualitative analyses examined emergent discussion themes and quantitative analyses compared feature ratings to determine which were perceived as most important.

**Results:**

Participants preferred a highly personalized and adaptive mHealth experience. Their ideal mHealth quit-smoking tool would allow personalized tracking of their progress, adaptively tailored feedback, and real-time peer support to help manage smoking cravings. Based on qualitative analysis of focus group discussion, participants preferred pull messages (ie, delivered upon request) over push messages (ie, sent automatically) and preferred interaction with other smokers through closed social networks. Preferences for entertaining games or other rewarding incentives to encourage program use differed by age group. Based on quantitative analysis of surveys, participants rated the importance of select design features significantly differently (*P*<.001). Design features rated as most important included personalized content, the ability to track one’s progress, and features designed to help manage nicotine withdrawal and medication side effects. Design features rated least important were quit-smoking videos and posting on social media. Communicating with stop-smoking experts was rated more important than communicating with family and friends about quitting (*P*=.03). Perceived importance of various design features varied by age, experience with technology, and frequency of smoking.

**Conclusions:**

Future mHealth cessation aids should be designed with an understanding of smokers’ needs and preferences for these tools. Doing so does not guarantee treatment effectiveness, but balancing user preferences with best-practice treatment considerations could enhance program adoption and improve treatment outcomes. Grounded in the perspectives of smokers, we identify several design considerations, which should be prioritized when designing future mHealth cessation tools and which warrant additional empirical validation.

## Introduction

Smoking is the leading preventable cause of disease and death in the United States [1] and kills 6 million people worldwide each year [2]. Despite this, an estimated 40 million people in the United States continue to smoke [3]. Most make a quit attempt each year [4], but do not use recommended behavioral or pharmacological treatments [5], preferring instead to quit on their own [6]. However, unaided cessation attempts are generally not effective [7]. Thus, there is an important need to create new nicotine dependence treatment programs and behavior change aids that are grounded in best-practice treatment recommendations, but are also acceptable and engaging to smokers. Well-designed tools that offer attractive intervention components (ie, design features) could enhance program utilization and improve treatment efficacy.

Advances in mobile computing offer the potential to deliver smoking cessation interventions that are both effective and engaging, in part because treatment can be delivered when and where it is needed. Research shows that technology-based interventions can effectively promote cessation [8-12], and can include a wide array of mobile health (mHealth) options ranging from wearable devices to sensors and mobile phone apps. Yet, beyond traditional text messaging, we know little about which mHealth intervention strategies are most promising. For example, apps are attractive to many smokers [13-15], but to date no large-scale randomized effectiveness trials of cessation apps have been published, only preliminary pilot studies with small samples or short follow-up periods [16-20] and protocols of trials in progress [21-24]. Among commercially available cessation apps, most fail to adhere to treatment guidelines [13-14], are not evidence based [15], and do not include design features that could most effectively support sustained behavior change [25], such as a personally tailored experience preferred by many smokers [26] and recommended by treatment experts [27].

It is too early to determine if mHealth smoking cessation apps or other tools will meet their potential. But it is clear that they will not if these programs are not designed with features that attract and engage smokers. In prior work, we showed that smokers and treatment experts do not always agree on design features that are most important [27]. In this paper, we advance the field with an in-depth characterization of smokers’ preferences and delineate key considerations that should be prioritized when designing mHealth cessation tools. Before describing our mixed-methods study, we summarize related work that motivates our focus on designing mHealth from the perspective of smokers.

### Approaching Design of mHealth Quit-Smoking Tools From the Perspective of Smokers

In the absence of empirical evidence to inform optimal mHealth program design, it is critical that clinical investigators and software developers consider the needs and preferences of smokers. This alone will not ensure treatment effectiveness, but it is an important consideration for treatment engagement, and engagement is necessary for efficacy. Surprisingly little published work has engaged smokers in the design process [28-30], yet important lessons can be learned from those who have. For example, Bock and colleagues [28] engaged smokers in the design of a text-based cessation program and learned smokers wanted a broader program that included social networking and control over the timing and delivery of text messages. Similarly, Ploderer and colleagues [30] field-tested a mobile app and discovered unanticipated strategies smokers use to cope with cravings and leverage peer support. Similarly, Paay and colleagues [29] engaged smokers in focus groups and design workshops and gained insight for tailoring smoking cessation apps to accommodate individual differences among smokers.

Other studies have not engaged smokers in the design process, but gained important information about smokers’ needs and preferences from observing their adoption and use of mHealth tools [31]. For instance, Heffner and colleagues [32] observed smokers’ use of a mobile quit-smoking app over 8 weeks and discovered that the most popular features were not necessarily associated with quitting. This underscores an important lesson—user preference does not guarantee treatment outcome. This is why it is important to ground design considerations in solid treatment theory and empirical evidence, but user preferences can offer important insights about how best to do this.

### Knowledge Gaps in Design of mHealth Quit-Smoking Tools

Solicitation of user preferences may be most effective when guided by existing knowledge gaps in the literature on messaging, social support, and incentivizing use. For example, it is known that text message reminders may be effective cessation aids [9,10] and may be useful for delivering tailored motivational messages [33,34], quit advice based on expert recommendations [10,35], and support on demand. However, text reminders about quitting smoking may also prompt people to smoke [29], so some smokers may not be receptive to this messaging. Some smokers prefer to control the timing of automated messages [28], whereas others prefer messages sent at unexpected times [35]. Smokers’ relative preferences for push messages received automatically versus pull messages delivered upon request has been largely unexplored [31], but is an important issue that warrants further exploration.

Similarly, social support is an important feature in smoking cessation programs [7,36]. Several technology-based strategies can be found in the literature, including mobile quit buddies [9], role model videos delivered via text message links [37], or public pledges posted on social media [15,38]. Research highlights unresolved tensions between the diffusion of quit-smoking information and experiences through online social networks [39-42] versus smokers’ desire to maintain anonymity [29,43]. These gaps also warrant further investigation to better understand how smokers wish to use mHealth technologies for social support during the quit process.

Finally, insight into smokers’ preferences for strategies that can make mHealth cessation aids rewarding and engaging can fill gaps in knowledge about how to best incentivize program use. Gamification is the use of game design elements in nongame contexts [44], such as health [45]. Some mHealth interventions pair game features with desired behaviors [46], such as encouraging physical activity by growing a digital garden as fitness goals are attained [47] or scoring points on familiar games (eg, hangman, Sudoku) for accurately estimating nutritional value of foods [48]. Gamification is well suited for motivational aspects of behavior change [49-51]. As a smoking cessation aid, game mechanics (eg, points, badges, leaderboards) have been used to encourage the use of educational “quit guides” [38]. Such game-based incentives can be integrated and viewable to others via social media [24,38]. However, others have advocated offering smokers more personally meaningful rewards, such as money [29]. Although prior work has elicited opinions of experts [52], smokers’ relative preferences for the use gamification features have been largely unexplored to date despite enthusiasm for their use by developers.

In summary, user-centered studies that engage smokers have proven valuable to uncovering important design considerations from the user’s perspective [28-30]. Given gaps in our understanding of smokers’ preferences for the range of possible mHealth support, further research is warranted to characterize their design priorities. In this study, we employed user-centered mixed methods to characterize the design preferences of smokers. Given the considerable opportunities to advance quit-smoking tools with mHealth innovations, such engagement of smokers in the design process is critical for identifying the most promising design features for future mHealth cessation tools.

## Methods

To characterize smokers’ design feature preferences for mHealth quit-smoking tools, we engaged smokers in a series of six focus groups using mixed methods, including group discussion and individual surveys. Our goals were to (1) qualitatively explore smokers’ “blue-sky” thinking (ie, brainstorming) about ideal mHealth design features; (2) elicit qualitative preferences among specific messaging, social support, and gamification features; and (3) quantitatively assess the importance of a selected list of features identified by treatment experts. Because preferences could differ by age, we completed three focus groups with younger adults aged 18 to 39 years and three focus groups with older adults aged 40 years and older. All data were collected in June of 2015 at Group Health Research Institute (GHRI). Study procedures were approved by the GHRI Institutional Review Board.

### Recruitment

We recruited smokers in the Seattle area via online ads (eg, Craigslist), posted flyers, and invitation letters mailed to likely smokers who were members of Group Health Cooperative (a nonprofit health care system in Washington State). Interested smokers were invited to contact study staff to learn more and be screened for eligibility. Individuals were eligible if they were at least 18 years old, a current smoker interested in quitting, could read and write in English, and owned a mobile phone or tablet computer which they used to access the Internet. We purposively recruited by age for three groups younger than 40 years of age and three groups 40 years of age and older.

### Procedures

Each focus group lasted approximately 2 hours and was moderated by the lead author (AH) with support and note taking by a coauthor (JB). We began each group with a paper survey to collect participant characteristics, including demographics (ie, race, ethnicity, education, employment status, income), smoking status (ie, cigarettes smoked in past 30 days, smoking frequency, plans to quit), and experience with technology (ie, some experience, intermediate, very experienced, expert). Then, participants engaged in a two-part group discussion. The first part focused on blue-sky brainstorming about the ideal design of future mHealth quit-smoking tools (part I). In the second part (part II), participants were asked to react to contrasting design options for mHealth messaging, social support, and incentivizing program use. At the end of the session, after participants had considered a range of design options for quit-smoking tools, they completed an exit survey rating their perceived importance of various mHealth features. Group discussion was audio recorded and transcribed for analysis, and field notes were written after each group meeting. Attendees received US $50 for their participation.

#### Part I: Blue-Sky Brainstorming

Inspired by the future workshop method [53], our goal was to generate visions for the ideal design of mHealth tools with desirable features for quitting smoking. Participants were asked: “If you were to design a mobile health tool to help you stop smoking, how would you want it to work? In a perfect world with no technical barriers, what features would you want such a tool to have?” We encouraged participants to consider a range of mobile devices (eg, mobile phones, tablets, wearables, sensors) and to share design ideas about content, interaction, and communication features in an open and nonjudgmental manner. We also encouraged participants to draw upon challenges in their prior experience with mHealth tools or attempts to quit smoking as they envisioned future designs that can address those barriers.

#### Part II: Preferences for Specific mHealth Features

We presented participants with two contrasting options for each of three design features: messaging, social support, and gamification. We selected these design features because they represent gaps in the literature. Messaging options for communicating with smokers were push messages that show up automatically on your device (eg, notifications, alerts, reminders) versus pull messages delivered only upon your request. Social support options were prerecorded peer testimonials delivered in the form of stories (eg, blogs or vlogs to passively view) about what helped others to quit smoking versus interactive social networking to actively interact with others and share information about yourself. Gamification options to promote use were entertaining games that are fun and distracting versus incentives that provide rewards, such as earning badges. We asked participants to discuss which option they preferred for each feature.

#### Exit Survey

At the close of the session, each participant completed an exit survey to rate the importance of 21 hypothetical app design features. Features were categorized into four domains: cost and reputation, privacy and security, content and user experience, and communication with others. Individual features were chosen to (1) reflect technology-based strategies for implementing best-practice treatment recommendations (eg, addressing use of pharmacotherapy, providing social support, and offering cognitive behavioral-based content), (2) reflect ways to leverage other mobile phone capacities to make these programs more engaging (eg, gaming), (3) assess perceived limitations of mHealth tools (eg, security and privacy), or (4) understand other user preferences which may inform future program development (eg, cost, reputation). Each feature was rated using a 4-point Likert scale (1=not at all important, 2=somewhat important, 3=very important, and 4=extremely important). Participants were asked to rate the importance of each feature if they were considering downloading or using a smoking cessation app.

### Analysis

We conducted a mixed-methods analysis of qualitative focus group transcripts and quantitative surveys. Following each focus group session, the two facilitators (AH, JB) met to summarize key themes that emerged. After completion of all six groups, the facilitators (who are both trained in qualitative interviewing and coding methods) reviewed field notes and applied thematic analysis [54] to transcripts to identify qualitative themes regarding participants’ blue-sky design ideas and preferences for messaging, social support, and gamification features of mHealth quit-smoking tools. Each facilitator summarized themes in transcripts, which were then reviewed by the second facilitator for accuracy. Any discrepancies were resolved during meetings between the coders. An initial synthesis of themes depicting the data were reviewed with study team members. This coding scheme was applied to individual transcripts by AH capturing representative quotes for the themes. Themes were compared across the six focus groups and between age groups.

We summarized participant surveys with descriptive statistics. We compared importance ratings among all design features with Friedman chi-square tests (χ^2^) and paired comparisons within domains with Wilcoxon signed rank tests (W). We compared ratings between younger and older participants and between participants grouped by experience with technology and smoking frequency with Mann-Whitney *U* tests (*U*). We adjusted *P* values for post hoc comparisons using a Bonferroni correction. We computed statistics in R (version 2.15.1) [55].

## Results

### Participants

Of the 119 respondents in total, 25 screened ineligible, one refused, and 38 could not be recontacted for eligibility screening. In all, 56 respondents were eligible and scheduled to attend a focus group stratified by age (<40 vs ≥40 years old); 16 failed to attend, leaving a final sample of 40 participants (Table 1). The younger focus groups included a total of 22 participants (P1-P22) and the older focus groups included a total of 18 participants (P23-P40). Participants were primarily white (63%, 25/40), non-Hispanic/Latino (90%, 36/40), and had less than a college degree (65%, 26/40). On average, participants smoked 12 cigarettes per day. Compared with younger participants, a higher proportion of older participants were heavy smokers. More than half of participants reported being “very experienced” with computers or better (58%, 23/40) and approximately half had health apps on their mobile phones (48%, 19/40). Examples of apps that emerged during group discussion focused largely on diet and fitness (eg, MyFitnessPal). Only five participants (13%) had downloaded a quit-smoking app before. Of the remaining 35 participants, the majority (83%, 29/35) reported they would consider downloading one.

**Table 1:** Participant characteristics by age group.

Characteristic		All (N=40)	Younger (<40 years) (n=22)	Older (≥40 years) (n=18)
**Age (years)**				
	Mean (SD)	38 (12)	29 (5)	50 (5)
	Range	20-58	20-39	40-58
Gender (female), n(%)		20 (50)	13 (59)	7 (39)
**Ethnicity, n (%)**				
	Hispanic/Latino	2 (5)	1 (5)	1 (5)
	Non-Hispanic/Latino	36 (90)	20 (90)	16 (90)
	Decline to state	2 (5)	1 (5)	1 (5)
**Race, n (%)**				
	White	25 (63)	15 (68)	10 (56)
	Black/African American	10 (25)	2 (9)	8 (44)
	Other/Multiple races	4 (10)	4 (18)	—
	Decline to state	1 (3)	1 (5)	—
**Education, n(%)**				
	Less than high school	3 (8)	3 (14)	—
	High school graduate	12 (30)	7 (31)	5 (28)
	Some college	11 (28)	3 (14)	8 (44)
	College graduate (BA, BS)	9 (23)	6 (27)	3 (17)
	Postgraduate (MA, MS, PhD, MD)	5 (13)	3 (14)	2 (11)
Cigarettes smoked per day in last 30 days, mean (SD)		12 (7)	9 (6)	14 (8)
**Smoking frequency, n (%)**				
	Light smokers (<10 cigarettes per day)	17 (43)	12 (55)	5 (28)
	Heavy smokers (≥10 cigarettes per day)	23 (58)	10 (45)	13 (72)
**Plans to quit smoking, n (%)**				
	I am not thinking about quitting smoking	1 (3)	1 (4)	—
	I am thinking about quitting smoking in the next 6 months	19 (48)	11 (50)	8 (44)
	I am thinking about quitting smoking in the next 30 days	7 (18)	5 (23)	2 (11)
	I am actively trying to quit smoking	12 (30)	5 (23)	7 (39)
	Decline to state	1 (3)	—	1 (6)
**Experience with technology, n (%)**				
	Some experience	3 (8)	1 (5)	2 (11)
	Intermediate	14 (35)	7 (32)	7 (39)
	Very experienced	20 (50)	11 (50)	9 (50)
	Expert	3 (8)	3 (14)	—
Have health apps on mobile phone, n (%)		19 (48)	12 (55)	7 (39)
Ever downloaded a quit-smoking app, n (%)		5 (13)	4 (18)	1 (6)

### Blue-Sky Design Ideas for Ideal mHealth Quit-Smoking Tools

Blue-sky brainstorming led to a range of design features smokers envisioned for future mHealth quit-smoking tools. Most participants expressed a strong overarching expectation for a highly individualized and responsive experience: “What if you had a profile where you could personalize it and make it your own?” (P1). Rather than follow a prescribed and impersonal quit process that several participants experienced with current apps, P12 wanted the ability to “...personalize your notification or whatever you want your phone to tell you. Write it yourself; set your own goals.” More specifically, the most universal design preferences that emerged across groups were personalized tracking with adaptive feedback and real-time peer support. Both of these themes reflect the critical importance participants placed on support to combat smoking cravings.

#### Personalized Tracking With Adaptive Feedback

When asked how they envisioned an ideal tool working in a perfect world, the first response from all groups was for immediate physical feedback on smoking behavior: “Maybe on a wearable it would be something that was like a little electric shock therapy thing. That’s pretty extreme, but...” (P10). Other examples included having the mobile phone or a bracelet “vibrate me” (P33). Another participant reflected: “I’m getting ready to smoke, okay, well I’m sending a shock to you, be prepared. Something a little more than talking is going to—yeah, they’re going to have to do some interference” (P22). Participants expressed a desire for immediate feedback to navigate smoking triggers and combat adaptation. For example, P33 expressed the concern: “After a while I’m going to see if I can get immune to that shock, something to build my tolerance to it just like we build our tolerance to nicotine now” (P33).

In addition to tracking smoking behavior, participants described concurrently tracking the context (eg, location) in which smoking occurs to intervene on triggers: “I could see one being kind of a diary...you would enter when you smoked and the environment you were in. And then it would kind of create like a profile for you, what your triggers are, and help you...just like my front page on my phone in the morning time gives me the news, this, that, and the other. ‘Hey [P33], let’s go to the gym instead of smoking that cigarette’” (P33). P35 added: “It would have to connect to your GPS” to make it easier to track your location and learn such patterns.

As discussion progressed, more sophisticated and nuanced design features emerged. These highlighted participants’ interest in personalized tracking that helps them navigate smoking triggers by giving behavioral feedback that adapts as a user cuts back and quits smoking. For instance, P8 described passively tracking smoking rhythms to predict and intervene before future triggers: “I have a pretty bad track record with manually keeping and tracking data...ideally with an app like that you would be able to give it a few parameters toward the beginning of it, things very generalized, like how much you smoke in a day, what cigarettes cost in your region, et cetera, and it would be able to sort of intelligently track metrics for you and reinforce that to you throughout time and that’s the encouragement to keep going.”

Younger participants were less willing than older participants to track triggers manually and tended to favor automatic capture of contextual information about triggers, such as through GPS location. However, some participants were uncomfortable with passive sensing: “I don’t like...knowing a computer is tracking where I’m going, so I usually leave those off if I can, the notifications of where you are at, because it’s kind of creepy” (P18). Some participants found value in manual tracking because it can raise behavioral awareness: “Half of the time we do it [smoking] when we’re not even thinking about it. But if we’re literally tracking consciously every time we did it...I think that would be—like you’re able to do it for a couple weeks, be like, ‘Wow!’” (P37). P36 agreed: “To get so when you’re having a cigarette it’s not an automatic mindless thing; that you’re really aware of what you’re doing and it’s a conscious decision that you’re making at that point.”

Coupled with personalized tracking, participants expressed the desire for adaptive feedback that matches their changing needs in two major ways. First, participants desired tailored feedback matched to their personal needs and interests. Some participants preferred receiving discouraging “scare tactics” (P36), such as pictures of black lungs, rather than encouragement. Other participants preferred feedback from a real person to automated messages. Most participants agreed that mHealth tools should enable users to select preferred styles of feedback from a menu of choices.

Second, the majority of participants desired dynamic feedback that varies and evolves as their smoking habits change. Adaptive feedback could “help you come up with a plan to reduce. What scares me is, like, quitting just cold turkey. So, like, a person’s specific plan to reduce day by day” (P11). In contrast, only one participant advocated for repetition in feedback over time: “If you hear it consistently, you know, you start thinking about it” (P26).

#### Real-Time Peer Support

A second universal theme that emerged in all groups was the design priority of real-time peer support. In contrast to ongoing tracking and feedback on smoking behavior, participants envisioned the ability to make emergent requests for support to combat smoking cravings. Although a few participants preferred a distracting image or encouraging message, most participants expressed value in connecting on demand with other smokers or people who had quit (ie, peers). For example, “You could have a small profile that you set up and choose somebody that’s almost like online dating but you’re just finding a quit partner” (P36).

Compared with peers, participants expressed little interest in connecting with their existing social networks (eg, family, friends) about their quit-smoking experience through social media they regularly use. When asked to compare real-time peer support with holding out for professional advice, one participant shared: “In the heat of a craving for me, it [advice from a health professional tomorrow] would probably be too late. I would prefer to connect with somebody right now while I’m having that craving and I’d prefer it be somebody in my area for some reason, just make it more personal” (P32). Several models of real-time pairings for peer support emerged, from a “quit-smoking buddy” (P35) to sponsors: “It’s like when somebody goes into AA [Alcoholics Anonymous] when they have that support…they have a sponsor they go to” (P28).

Other participants envisioned design features that tap into a larger support network through video calls, voice, or text: “If there’s a big red button, ‘Oh, I’m about to smoke’ and I hit it and then like Chatroulette it randomly picked me with someone” (P9). Another participant envisioned “a ‘911’ [emergency hotline] and then you just get on your phone, hit that app, and automatically it will dial into like a support system, like a hotline or a person. And you say, ‘Oh, I’m ready to smoke, is there something I can do?’ And that person will give you suggestions to steer you away” (P28). Because cravings can come on strong and last several minutes, some participants thought automated support might be more feasible than having a real person “on call” 24/7. However, many participants thought that interacting with a real person could be essential for not giving in to cravings.

Participants also envisioned tools for exchanging quit-smoking advice within a social network of peers. For example, P9 described “a blog space where you have the availability to see tips and tools that have helped other people” (P9). Another participant envisioned “a club for quitting smoking through this club on your phone, this app” (P24). In one focus group session, a participant suggested using GPS to connect “in your very own city if you need to meet up to talk about it” (P3). However, participants agreed that meeting strangers face-to-face could raise privacy and security concerns. Yet more generally, younger and older participants agreed that real-time peer support delivered safely is a key design priority for mHealth quit-smoking tools.

### Design Preferences for Messaging, Social Support, and Gamification

Participants’ preferred design options expanded on themes from their blue-sky ideation of mHealth quit-smoking tools. Their feedback on preferred options for messaging, social support, and gamification features provided deeper insight into their needs and priorities as target users.

#### Messaging

Compared with “push messages” sent automatically, most participants preferred “pull messages” they could request in real time, especially to help manage emergent cravings to smoke. Participants varied in the type of messages they wished to pull, ranging from photos of loved ones to chat with peers. Messages that were personalized and reinforcing were preferred by a majority of participants. For example, P5 suggested “a note to yourself, a kind of personalized quit plan” and P36 suggested “affirmations or telling you how much your lung capacity has improved.”

Several participants described their distaste for automated alerts and push notifications that they routinely disable on their mobile phones. Participants expressed concern that poorly timed push messages would only remind them about smoking and trigger cravings. One participant worried that push messages for quitting smoking might be too easy to dismiss: “You have to combat it [a craving] with something that’s going to be more foolproof [than a notification] because it is a very strong habit” (P24). A few participants expressed interest in receiving infrequent, but targeted notifications if they could control timing, frequency, and delivery. Examples included messages timed with cravings, such as alternatives to smoking (“Instead of smoking a cigarette, eat a banana” P35), encouragement (“You’ve gone 3 hours without smoking, congratulations” P27), or reminders (“Go to the gym instead of smoking that cigarette” P33). One participant expressed interest in receiving messages from a quit-smoking “sponsor” (P28).

Although concern was raised about timing push messages to coincide with cravings, a few participants reflected on the opportunity if push messages were adaptively coupled with intelligent tracking features: “I think it would be extraordinarily difficult...with some sort of automated message, it would have to be dialed in pretty deep to gain the proper context [of a craving]. Push notifications on the other hand, based off of data that you actually initially give the app—and that’s something maybe you would be able to give over time rather than having to keep regular track of any sort of data” (P1). P8 agreed: “Being able to set notifications based on either time or geography based off like GPS location or an address—I can see that being really, really useful” (P8).

#### Social Support

Compared with social support from peer testimonials in the form of stories that could be read (eg, blog), listened to (ie, audio), or watched (ie, video), most participants preferred interactive social network features, such as making friend connections with other smokers, sharing user profiles, and exchanging messages. Echoing the priority of peer support during brainstorming, the majority of participants expressed the most interest in a closed social network of similar peers: “Some sort of community of just like-minded people [who] were there for one common goal regardless of where they are from [sic]. That needs to be the only similarity really, is that trying to quit. This is what we are here for” (P19).

Although a few participants expressed interest in supportive connections with their spouse or health care provider, most preferred greater anonymity: “A lot of times you will be quitting smoking, you will tell all your friends because you want them to encourage you, and it’s like your double-edged sword when you are doing well, but when you are not you have this guilt associated with it. I am not going to tell anybody because if I fail. But if you have people encouraging you, they are not going to care if you fail, but they are going to be encouraging you along the way, so it’s the support without the personal attachment” (P22). A few participants expressed interest in connecting with a quit-smoking sponsor who had previously quit or health care professionals to whom they might not otherwise have access. A couple of participants were interested in connecting with counselors or quit coaches through a mHealth quit-smoking tool.

One participant saw value in “testimonials from people that have quit of like the things that they didn’t realize that were hidden benefits [of quitting]” (P22). However, most participants found peer testimonials less appealing than social networking because prerecorded stories can be impersonal: “If it’s just story after story, I’m this person, I smoked for...I would get bored really easy. If they were more like tell a brief story and here’s an exercise routine, and they walk you through it. I would be more inclined to stay and listen” (P3). Overall, younger and older participants were interested in tips and advice from peers who had successfully quit, but sought interactive modes of communication, such as a discussion forum.

#### Gamification

Given the choice, participants did not have a clear preference for either entertaining games or rewarding incentives based on program use; rather, participants preferred aspects of both. Older participants found traditional incentives and rewards more appealing, whereas younger participants described more interest in and diversity of gaming features.

Younger participants described a broad number of gaming features of interest, but mostly, participants preferred games that could serve as fun distractions from cravings to smoke. Examples ranged from simple distractors, such as the popular clicker app “cow evolution” (P19), to more involved role-playing or social games. For example, P22 told us: “I see sort of like a cast of characters, the good cop and bad cop, the stop-smoking buddy, the drill sergeant, the temptress so to speak to try to vanquish—‘No, no, I shall not smoke’—some sort of little Sims-type world.” Other participants described interacting with other users through social gaming features: “[This discussion] makes me think of a ‘swarm’ on Foursquare, like when people are all in the same place...adding experience points and leveling up and achievements that you’re all basically earning extra credit for” (P8). Social games were thought to stimulate “a healthy sense of competition” (P8) and remind users “you’re not the only one struggling” (P22).

Several younger participants talked about incorporating rewards into games such as “leveling up.” Yet a broader range of rewards that could help smokers stay motivated to quit surfaced in all groups, both extrinsic and intrinsic incentives. For example, participants in every group talked about loyalty points and monetary rewards: “A money reward...it’s not to be paid [to quit smoking]...it’s an added extra—it’s a reward” (P28). Other extrinsic rewards included personal accountability to others, such as sharing progress with a quit-smoking sponsor or buddy: “You want to input your progress too so other people can track what you’re doing and they can motivate you, say, ‘keep it up,’ you know. That will keep you going. So you don’t want to let them down” (P35).

Intrinsic rewards included a feature “that reminds me of the progress I make” (P12) or enables one to “challenge yourself” (P3). A couple of participants suggested tying the design to behavior change theory: “There’s like a bunch of different stages of change, and they could measure where you’re at and kind of work with you to see what has to be done to get you to the next level” (P5).

### Importance Ratings for Select mHealth Features

Following focus group discussion that covered a wide range of design ideas and options, participants completed the exit survey to individually rate the importance of 21 specific design features (Table 2), each chosen to reflect best-practice treatment, ways to leverage mobile technology to support quitting smoking, or other important user preferences across domains of cost and reputation, privacy and security, content and user experience, and communication [27].

**Table 2:** Perceived importance of mHealth design features using 4-point Likert ratings.^a^

Design feature		All, mean (SD) (N=40)	Younger, mean (SD) (n=22)	Older, mean (SD) (n=18)
**Cost and reputation: a tool that...**				
	Is free or low cost	3.4 (0.8)	3.3 (0.8)	3.4 (0.7)
	Is highly rated by other people	2.8 (1.0)	2.7 (1.0)	2.9 (1.0)
	Is “research tested”	2.8 (0.9)	2.7 (0.9)	2.9 (0.9)
	Is endorsed by clinical experts	2.7 (1.0)	2.5 (0.9)	2.9 (1.1)
**Privacy and security: a tool that...**				
	Keeps your information private	3.3 (0.8)	3.1 (0.9)	3.6 (0.7)
	Stores information on your phone	2.8 (1.0)	2.4 (1.0)	3.2 (0.9)
	Stores information in a secure “cloud”	2.6 (1.2)	2.4 (1.1)	2.9 (1.2)
**Content and user experience: a tool that...**				
	Includes games or entertainment	2.5 (1.0)	2.7 (1.0)	2.2 (0.9)
	Matches content to your personal needs and interests	3.5 (0.6)	3.6 (0.6)	3.4 (0.7)
	Changes content as your needs and interests change	3.2 (0.9)	3.4 (0.8)	3.0 (1.0)
	Helps you manage nicotine withdrawal or medication side effects	3.5 (0.6)	3.5 (0.7)	3.5 (0.6)
	Helps you track your progress (cigarettes/day)	3.5 (0.7)	3.6 (0.5)	3.3 (0.9)
	Sends supportive or motivational messages (eg, text or email)	2.8 (1.0)	2.4 (1.0)	3.2 (0.9)
	Includes stories from other smokers about quitting	2.6 (0.9)	2.5 (0.9)	2.7 (1.0)
	Includes videos about quitting smoking	2.1 (1.0)	1.8 (0.9)	2.3 (1.1)
	Includes information on stop-smoking medicines	2.6 (1.1)	2.3 (1.0)	2.9 (1.1)
**Communication: a tool that...**				
	Lets you to communicate with other smokers about your progress	2.8 (1.0)	2.6 (1.0)	3.1 (1.1)
	Lets you communicate with family and friends about your progress	2.4 (1.1)	2.1 (1.0)	2.6 (1.1)
	Lets you communicate with stop-smoking experts about your progress	2.9 (0.9)	2.6 (1.0)	3.2 (0.8)
	Lets you communicate with your personal doctor or health care team	2.5 (1.0)	2.3 (1.0)	2.8 (1.0)
	Lets you post information on Facebook or other social media sites	1.8 (1.0)	1.7 (0.9)	2.0 (1.1)

^a^ 1=not at all important, 2=somewhat important, 3=very important, and 4=extremely important.

Participants rated some design features as significantly more important than others (χ^2^=189, *P*<.001). The highest ratings (n=40) were for tools that matched content to ones needs and interests, helped with withdrawal and side effects, and tracked progress. The lowest rated feature for all was posting to social media. This pattern was consistent across age groups, with the exception of older participants placing the greatest importance on privacy.

For readers interested in a granular examination of how perspectives on the 21 surveyed design features differed, we include a full list of paired comparisons within domains by each age group in the Multimedia Appendix 1. Briefly, importance ratings differed significantly within each domain (cost and reputation: χ^2^_3_=21.2, *P*<.001; privacy and security: χ^2^_2_=11.6, *P*=.003; content and user experience: χ^2^_8_=96.1, *P*<.001; communication: χ^2^_4_=46.8, *P*<.001). Paired comparisons suggested participants prioritized cost, maintaining privacy, and the ability to track, obtain personalized content, and support for nicotine withdrawal over other design features in mHealth tools. Participants also prioritized communicating about smoking outside of social media, perhaps with individuals whom are less familiar. For example, ratings were significantly higher for communicating with stop-smoking experts than family and friends (W=38, *P*=.03). Further, several participants shared the sentiment expressed by P32: “It’s a lot easier to be honest with somebody that you don’t personally know because like my doctor I’ve had her for 15 years and I love her, yet there’s stuff I don’t want her to know. But I want to be able to be honest at the same time.”

Compared with younger participants, older participants rated the following features as significantly more important: a tool that “stores information on your phone” (*U*=116, *P*=.02), “sends supportive or motivational messages” (*U*=113, *P*=.02), and “lets you communicate with stop-smoking experts about your progress” (*U*=122, *P*=.03). Compared with participants who had some or intermediate experience with technology (n=17), those who were very experienced or expert (n=23) rated “helps you track your progress” significantly more important (*U*=113, *P*=.01).

Compared with heavier smokers (n=23), lighter smokers (n=17) rated the importance of most features higher overall. Lighter smokers rated the following features significantly higher than heavier smokers: a tool that “matches content to your personal needs and interests” (*U*=250, *P*=.03) and “changes content as your needs and interests change” (*U*=271, *P*=.03). However, both of these features were among the features heavier smokers rated highest. There were no significant differences in importance of features rated by participants who were actively trying or thinking of quitting in the next 30 days (n=19) and participants thinking about quitting in 6 months (n=19).

Finally, after synthesizing the feedback from both the qualitative focus group feedback and qualitative survey results, several key design considerations emerged and are presented in Table 3.

**Table 3:** Synthesis of preferred design considerations across assessment methods.

Design consideration	Part I: qualitative blue-sky brainstorm	Part II: qualitative design preferences	Exit survey: quantitative importance ratings
Content and user experience	Support personalized tracking of smoking behavior and context (eg, location) with tailored, dynamic feedback that adapts to evolving needs	Enable pull messages on demand with personalized content; provide distracting games, social games, and extrinsic or intrinsic rewards	Enable users to track progress on quitting; offer personalized content; provide support for nicotine withdrawal
Communication channels for support	Enable real-time peer support to combat smoking cravings and exchange quit smoking advice	Offer a closed network to connect and interact with current and ex-smokers about quitting	Protect smokers from exposing personal smoking information on social media; help smokers connect with experts
Other key considerations	Create a highly personalized and responsive support through active and passive channels	Target select features to groups based on preference (eg, gaming for younger smokers)	Offer tools for free or low cost; keep information private

## Discussion

### Principal Findings

Using a mixed-methods approach, we explored smokers’ ideal design features for mHealth cessation tools and assessed the relative importance of specific design considerations and treatment components that are particularly relevant to this design space. Our qualitative findings illuminated a number of creative design ideas, but most importantly, highlighted smokers’ desire for a highly personalized and adaptive experience, the ability to connect with peers for support, and the use of active and passive communication channels. Qualitative insights also highlighted preference for pull messages (ie, delivered upon request) over push messages (ie, sent automatically), interaction with other smokers through closed social networks, and targeted incentives based on user group. Quantitative results reinforce considerations of importance, including personalized content, the ability to track progress, support for nicotine withdrawal, connecting with peers and quit-smoking experts, and keeping information private. However, some preferences vary by age, experience with technology, or smoking frequency. For example, older smokers were more sensitized to issues of privacy and younger smokers were more interested in gaming as a means of smoking distraction. Smokers who had more experience with technology placed more importance on tracking features than those with less experience. Compared with heavy smokers, lighter smokers placed more importance on personalized content that matches and dynamically adapts to one’s changing needs and interest.

Although incorporating smokers’ preferences will not guarantee mHealth tools will be effective, balancing user preferences with best-practice treatment considerations could enhance program adoption and improve treatment outcomes. Thus, these findings offer guidance to addiction treatment experts and developers working in this design space.

### Implications for Future Research and Development

The results of this study have implications for future research and development. For example, smokers wanted a highly personalized experience that included tracking (of smoking and smoking locations) and adaptively tailored content. In the future, tracking tools could couple self-monitoring [29] with passive sensing via wearable devices [56,57] and machine learning to predict smoking triggers and proactively intervene with real-time tailored recommendations [58]. This intelligent tracking should coincide with smokers’ cravings and deliver support when and where it is needed [59]. Notably, this type of passive push intervention is in contrast to smokers’ stated preference for active pull messaging to control timing on demand [28,31]. Remaining unanswered empirical questions include which type of intervention smokers would actually prefer based on real-world experience and how effective each strategy is relative to the other. These are important issues for future research.

Another design consideration worthy of further exploration is the use of gaming features in mHealth cessation programs. Relative to other design features, few studies to date have employed gamification strategies to help people quit smoking [24,38,52], but one could envision these strategies being used to incentivize participation or reaching milestones (eg, 48 hours smoke-free) using earned badges or offering game play to distract smokers during cravings to smoke. Among our participants, older smokers were more responsive to traditional rewards and incentives [29,60,61], whereas younger smokers were more open to gaming features. The use of gaming features have also been recommended by design experts [52,62], but further research is needed to inform whether these features can truly enhance treatment participation or outcome and for whom.

Social support from peers was also highly valued by our participants, as was support from stop-smoking experts. Both of these are recognized as important best-practice treatment components [7], so understanding how to most effectively integrate this support in mHealth interventions is another important consideration for future research. Our findings suggest that smokers want support from peers “on demand” and most preferred interactive modes of support over prerecorded peer testimonials, such as automated video messaging found ineffective for cessation in prior work [37]. Participants’ lack of interest in posting to social media echoes patients’ preferences for sharing personal health information on closed online networks that are condition-specific rather than general-purpose social media they regularly use [63]. However, with experience, smokers might find value posting in quit-smoking communities [64-66], particularly if combined with interventions tailored to one’s readiness to quit [67]. Open research questions remain about protecting privacy [64,66,68] while facilitating exchanges among social ties through which smoking cessation information is known to spread [39-42].

Our results further suggest communication with treatment experts may be more highly valued than sharing one’s progress with friends, family, or even a personal doctor. Congruent with prior research [29], these findings may reflect a preference for the anonymity of weak social ties (ie, peer smokers, treatment experts) over strong social ties. If so, this preference for greater anonymity would support the notion that smokers are “ambivalent socializers” who are simultaneously keen yet reluctant to engage with others about smoking via social media [68]. Yet this preference could create a treatment challenge. Support provided by a trusted clinician through counseling is an important element of best-practice cognitive behavioral-based nicotine dependence treatment [7], in part because it holds smokers accountable. Interacting with others anonymously could reduce the risk of peer pressure or embarrassment due to failures to quit [64], but could also reduce the effectiveness of the intervention due to lack of accountability. This raises yet another important area for future research to determine the effectiveness of anonymous peer support and expert advice provided in the context of mHealth cessation interventions.

### Strengths and Limitations

Strengths of this study include a diverse demographic group and our mixed-method research design. The use of blue-sky thinking to illuminate smokers’ “perfect world” design priorities is a unique strength of this paper. To our knowledge, this approach has not been used previously in this context. It revealed many creative design ideas, some of which were further confirmed as important in participants’ quantitative survey ratings. Finally, our comparison of younger versus older smoker preferences highlighted important distinctions in these groups and is another strength of the study design.

The study also has some limitations. First, the findings may not generalize to all smokers due to the small sample size. Participants varied in age, education, experience with technology, and smoking frequency, yet their limited ethnic and racial diversity may have not captured opinions across known differences in smoking patterns [69]. It is also possible that the views represented do not generalize to smokers who do not own mobile devices, although the opinions of that group are less relevant in this context.

Finally, our results only reflect the perceived preferences of smokers’ for mHealth cessation tools. Future research is needed to determine if these stated preferences hold true in real-world practice. Such studies could lay the foundation for future work to assess the relative value and effectiveness of mHealth tools compared with other quit-smoking modalities (eg, peer support groups, professional behavioral support, pharmaceuticals, and nicotine replacement).

### Conclusions

We assessed smokers’ design preferences for future mHealth smoking cessation tools and identified several features, which should be prioritized when designing future mHealth cessation tools. These include making programs that are highly personalized, adaptive, interactive, and can facilitate communication with peers and experts. Prioritizing these features in future mHealth interventions could improve their acceptability to smokers and program engagement, and improve treatment effectiveness as a result. However, the integration of popular mHealth features alone may not ensure a program’s use or effectiveness [32]. Future programs must also be grounded in relevant behavioral theory [59] and evidence-based treatment recommendations [13-15]. Aligning design preferences with evidence-based solutions is particularly important for addictive behaviors such as smoking [70] in which design priorities of treatment experts and smokers can differ [27].

Future mHealth smoking cessation tools will also require empirical validation. We highlight several important research questions worthy of investigation. It is our hope that this work will inform and inspire collaboration between addiction treatment experts, designers, and developers to create the next generation of mHealth smoking cessation tools.

## Appendix

Multimedia Appendix 1

## Abbreviations

GHRI: Group Health Research Institute
mHealth: mobile health
