# Supplementary material for: Prioritizing the mHealth Design Space: A Mixed-Methods Analysis of Smokers’ Perspectives
Source: JMIR Mhealth Uhealth. 2016 Aug 5;4(3):e95. doi: 10.2196/mhealth.5742 (PMC4992168; doi:10.2196/mhealth.5742)
Supplement: Multimedia Appendix 1 [file mhealth_v4i3e95_app1.pdf]

Appendix. Adjusted p-values for paired comparisons (V) of importance ratings for design features by domain for all participants (A), younger participants (B), and older participants (C). Significant differences (*P*<.05) are bolded.

[illegible] participants

COST & REPUTATION

A tool that ...

	Is free or low cost	Is highly rated by other people	Is "research tested"	Is endorsed by clinical experts
Is free or low cost	-	**.036**	**.012**	**.012**
Is highly rated by other people	-	-	1.00	1.00
Is "research tested"	-	-	-	1.00
Is endorsed by clinical experts	-	-	-	-

COST & REPUTATION

	Is free or low cost	Is highly rated by other people	Is "research tested"	Is endorsed by clinical experts
Is free or low cost	-	[illegible]	[illegible]	[illegible]
Is highly rated by other people	-	-	[illegible]	[illegible]
Is "research tested"	-	-	-	1.00
Is endorsed by clinical experts	-	-	-	-

PRIVACY & SECURITY

	Keeps your information private	Stores information on your phone	Stores information in a secure "cloud"
Keeps your information private	-	**.009**	**.010**
Stores information on your phone	-	-	1.00
Stores information in a secure "cloud"	-	-	-

(**All participants, cont**)

PRIVACY & SECURITY

	Keeps your information private	Stores information on your phone	Stores information in a secure "cloud"
Keeps your information private	-	.081	.168
Stores information on your phone	-	[illegible]	[illegible]
Stores information in a secure "cloud"	-	-	-

CONTENT & USER EXPERIENCE

A tool that ...

(**Older participants, cont**)

(**Younger participants, cont**)

	Includes games or entertainment	Matches content to personal interests	Changes content with needs	Helps with nicotine withdrawal	Helps you track progress	Sends supportive messages	Includes stories from other smokers	Includes stop-smoking videos	Includes stop-smoking medicine info
Includes games or entertainment	-	**<.001**	**.036**	**.004**	**.004**	1.00	1.00	1.00	1.00
Matches content to personal interests	-	-	1.00	1.00	1.00	**.036**	**.001**	**<.001**	**.001**
Changes content with needs	-	-	-	1.00	1.00	.720	.114	**.001**	.108
Helps with nicotine withdrawal	-	-	-	-	1.00	**.036**	**.001**	**<.001**	**.001**
Helps you track progress	-	-	-	-	-	**.036**	**.001**	**<.001**	**.004**
Sends supportive messages	-	-	-	-	-	-	1.00	.108	1.00

CONTENT & USER EXPERIENCE

	Includes games or entertainment	Matches content to personal interests	Changes content with needs	Helps with nicotine withdrawal	Helps you track progress	Sends supportive messages	Includes stories from other smokers	Includes stop-smoking videos	Includes stop-smoking medicine info
Includes games or entertainment	-	[illegible]	[illegible]	[illegible]	[illegible]	[illegible]	1.00	[illegible]	1.00
Matches content to personal interests	-	-	1.00	1.00	1.00	[illegible]	[illegible]	[illegible]	[illegible]
Changes content with needs	-	-	-	1.00	1.00	[illegible]	[illegible]	[illegible]	[illegible]
Helps with nicotine withdrawal	-	-	-	-	1.00	[illegible]	[illegible]	[illegible]	.072
Helps you track progress	-	-	-	-	-	[illegible]	[illegible]	[illegible]	[illegible]
Sends supportive messages	-	-	-	-	-	-	1.00	1.00	1.00
Includes stories from other smokers	-	-	-	-	-	-	-	[illegible]	1.00
Includes stop-smoking videos	-	-	-	-	-	-	-	-	[illegible]
Includes stop-smoking medicine info	-	-	-	-	-	-	-	-	-

COMMUNICATION

A tool that lets you...

	Communicate with other smokers	Communicate with family and friends	Communicate with stop smoking experts	Communicate with personal doctor	Post information on social media
Communicate with other smokers	-	.830	1.00	1.00	.046
Communicate with family and friends	-	[illegible]	[illegible]	[illegible]	[illegible]
